# Sulphate of Zinc as a Specific in Scarlet Fever

**Published:** 1883-03-20

**Authors:** W. D. Hoyt

**Affiliations:** Rome, Georgia


### SULPHATE OF ZINC AS A SPECIFIC IN SCARLET FEVER.

BY DR. W. D. HOYT, OF ROME, GEORGIA.

Whilst talking with Dr. C. S. Harris, a retired physician of this county, some months since, he gave me the following account of his use of sulphate of zinc in scarlet fever:

He stated that, whilst practicing medicine some years before in another part of the State, he encountered an epidemic of this disease which was quite fatal; that he had under his charge a patient, a little girl about eight years of age, who was suffering from so violent an attack of this disease that he anticipated unfavorable termination; that he had notified the relatives of his gloomy prognosis; received from them a request that he would call in their old family physician from another part of the State, who chanced to be in the neighborhood. This he gladly did. After visiting the patient, Dr. Harris remarked to the consulting physician, "That case is bound to die." "I see no need for it," replies the other. "What," says Dr. Harris, "do you know any remedy which will counteract this disease?" "I do," says the other; "If you will rub nine grains of zinc into a fine powder, divide it into nine powders, and give one every third hour, you will find her better to-morrow." Dr. Harris acted on this suggestion, but at his next visit was surprised at the improvement. The interval between the doses was lengthened to four hours, and the numbers finally reduced to three a day. Under this treatment the child rapidly recovered. Since this time the Doctor has used this remedy, he says, in numerous cases, and always with the happiest results. He has no theory as to the action of the remedy, but simply knows it cures. In no case, he states, has he seen it produce emesis or other unpleasant results.

On the 13th of April, 1883, I was called to see Lula McIntosh, a little girl four or five years of age, who had been taken sick the day before with high fever, headache, stupor and pain in the back and limbs. She presented, at my visit, the following symptoms. From head to foot the most distinct and intense scarlatinous eruption presented itself. The tongue was heavily coated; red and enlarged papillæ, showing through the white coat. The throat was redder than natural, but was not sore; pulse, 160; temperature, 104. There was no scarlet fever prevailing; but some relatives had arrived at the house from Tennessee some ten days before, and it was thought they had introduced the disease. It certainly seemed to me to be scarlet fever, in spite of the absence of throat symptoms. I was desirous of trying Dr. Harris' remedy, but was afraid of the dose. I, therefore, ordered 1-18th of a grain of sulphate of zinc, rubbed up with sugar, to be given every three hours, and ordered her thoroughly greased.

On the 14th I requested Dr. J. B. S. Holmes to see the case with me. He, also, was of the opinion that it was scarlet fever. The child had improved very much. The eruption had almost entirely disappeared; the tongue had cleansed; looked like a piece of raw beef. Pulse was 101; temperature, 101¾. The child felt well; had felt better, she said, after the second dose of medicine. She wanted food and wanted to get up.

On the 15th the child had still further improved—pulse, 128; temperature, 100.

On the 16th, pulse 120; temperature, 98½.

On the 18th desquamation had commenced, and an epithelial coat was being formed on the tongue. The bowels had been acted on every day; no sickness had been produced. The medicine was gradually diminished after the 16th.

Scarlet fever is, at least of late years, so rare a disease in Georgia that it may be a long time before I encounter another case in which to try the remedy. I, therefore, write this article, based on a single case, in order to request other physicians, more likely to encounter the disease, to test the efficacy of sulphate of zinc in scarlet fever. If it is the specific which Dr. Harris claims for it, its value cannot be over-estimated.—*Nashville Jour. of Med. and Surgery.*

